# Effects of a *Bacillus*-based direct-fed microbial on in vitro nutrient digestibility of forage and high-starch concentrate substrates

**DOI:** 10.1093/tas/txac067

**Published:** 2022-05-17

**Authors:** Liyi Pan, Karen Harper, Oscar Queiroz, Giuseppe Copani, Bruno I Cappellozza

**Affiliations:** University of Queensland, School of Agriculture and Food Sciences, Gatton 4343, Australia; University of Queensland, School of Agriculture and Food Sciences, Gatton 4343, Australia; Chr. Hansen A/S, Hørsholm 2970, Denmark; Chr. Hansen A/S, Hørsholm 2970, Denmark; Chr. Hansen A/S, Hørsholm 2970, Denmark

**Keywords:** *Bacillus licheniformis*, *Bacillus subtilis*, digestibility, forage, in vitro, starch

## Abstract

Two experiments evaluated the effects of a *Bacillus*-based direct-fed microbial (**DFM**) on in vitro dry matter (**DM**) and neutral detergent fiber (**NDF**; experiment 1) and starch (experiment 2) digestibility of a variety of ruminant feedstuffs. In experiment 1, 10 forage sources were evaluated: ryegrass, alfalfa hay, leucaena, corn silage, spinifex, buffel grass, flinders grass, Mitchell grass, Rhodes grass hay, and Queensland bluegrass. Experimental treatments were control (forages with no probiotic inoculation; **CON**) and forage sources inoculated with a mixture containing *Bacillus licheniformis* and *Bacillus subtilis* (3.2 × 10^9^ CFU per g; DFM). In vitro DM and NDF digestibility were evaluated at 24- and 48-h post-treatment inoculation. Treatment × hour interactions were noted for IVDMD (in vitro dry matter digestibility) and IVNDFD (in vitro neutral detergent fibre digestibility) (*P* ≤ 0.05). More specifically, DFM inoculation increased (*P* ≤ 0.03) IVDMD at 24 h in four forages and increased 48-h IVDMD (*P* ≤ 0.02) in alfalfa hay, ryegrass, leucaena, and Mitchell grass hay, but opposite results were observed for Queensland bluegrass (*P* < 0.01). A 24- and 48-h IVNDFD increased following DFM inoculation (*P* ≤ 0.02) in five forage sources, but reduced for Queensland bluegrass (*P* < 0.01). When the forages were classified according to their quality, main treatment effects were detected for IVDMD (*P* ≤ 0.02) and IVNDFD (*P* < 0.01). In experiment 2, five common cereal grains were evaluated—high-density barley (82 g/100 mL), low-density barley (69 g/100 mL), corn, sorghum, and wheat—under the same treatments as in experiment 1. In vitro starch digestibility (**IVSD**) was evaluated at 6- and 12-h following treatment inoculation. Treatment × hour interactions were observed for starch digestibility in three out of five concentrate sources (*P* ≤ 0.001). Inoculation of DFM yielded greater 24-h starch digestibility for high-, low-density barley, and wheat (*P* ≤ 0.02), but also greater at 48 h in wheat (*P* < 0.0001). Moreover, mean starch digestibility improved for corn and sorghum inoculated with DFM (*P* < 0.01). Using a *Bacillus*-based DFM (*B. licheniformis* and *B. subtilis*) improved the mean in vitro DM and NDF digestibility of different forage sources of varying qualities (based on crude protein content). Similarly, IVSD was also greater following DFM inoculation, highlighting the potential of this probiotic to improve nutrient digestibility and utilization in the beef and dairy cattle herd.

## INTRODUCTION

There has been an increasing interest in the utilization of direct-fed microbials (**DFM**) in beef and dairy cattle diets, as potential alternatives to antibiotic feeding (Krehbiel et al., 2003). Per definition, DFM, or probiotics, are classified as live microorganisms that, when administered in adequate amounts, confer health benefits to the host (FAO/WHO, 2001). Among the strains of probiotics often evaluated for ruminants, *Lactobacillus* spp., *Saccharomyces cerevisiae*, and *Bifidobacterium* spp. are highlighted, but attention to other strains is also given. One such strain is *Bacillus* spp., which has been currently used as probiotics (Luise et al., 2022) and has a wide range of applications and stability, allowing it to be included in different ruminant supplements.

Bacilli are Gram-positive, spore-forming, aerobic, and facultative anaerobic bacteria (Bernardeau et al., 2017), and currently more than 2,700 species of *Bacillus* spp. have been recognized (www.lpsn.dsmz.de). Recently, Luise et al. (2022) reviewed a series of experiments that highlighted the potential benefits of *Bacillus* spp. on the health and performance of monogastric animals, including direct and indirect pathogen inhibition, immunostimulatory effects, and nutrient digestibility and utilization. In fact, earlier studies reported that several *Bacillus* spp. can produce a wide set of fibrolytic, amylolytic, lipolytic, and proteolytic enzymes (Ghani et al., 2013; Elshaghabee et al., 2017; Su et al., 2020) that might enhance nutrient digestibility and performance of the animals. To the best of our knowledge, no other research trial evaluated the effects of *Bacillus* spp. on in vitro digestibility of forages and concentrates often included in ruminant diets. Hence, it was hypothesized that the inclusion of a combination of *Bacillus* spp. would improve in vitro fiber and starch digestibility of different substrates. Our objective was to evaluate the effects of a DFM-*Bacillus* base product on in vitro fiber (experiment 1) and starch (experiment 2) digestibility of different ruminant feedstuffs.

## MATERIALS AND METHODS

### Experiment 1: Forage Sources

This experiment was conducted at the University of Queensland (Gatton campus) from May to August 2021.

A total of 10 forage sources originated from Australia were evaluated in the present study: ryegrass (*Lolium* spp.), alfalfa hay (*Medicago sativa*), leucaena (*Leucaena leucocephala*), corn silage (*Zea mays*), spinifex (*Geophaps plumifera*), buffel grass (*Cenchrus ciliaris*), flinders grass (*Iseilema*), Mitchell grass (*Astrebla*), rhodes grass hay (*Chloris gayana*), and Queensland bluegrass (*Dichanthium sericeum*). All forage sources were analyzed in duplicates by wet chemistry procedures for concentrations of crude protein (**CP**; method 984.13; AOAC, 2006), neutral detergent fiber (**aNDFom**; Van Soest et al., 1991; modified for use in an Ankom-200 fiber analyzer; Ankom Technology Corp., Fairport, NY), acid detergent fiber (ADFom; method 973.18 modified for use in an Ankom-200 fiber analyzer; Ankom Technology Corp.; AOAC, 2006), and starch for corn silage only (Bach Knudsen, 1997). Moreover, total digestible nutrient concentration was calculated according to the equations proposed by Holland and Kezar (1995). The nutritional profile of the substrates evaluated herein is presented in Table 1.

**Table 1.** Nutritional profile of the forages and concentrate sources used in experiment 1 and 2, respectively

Item	DM, %^1^	% DM				
		CP	aNDFom	ADFom	Starch	TDN^2^
Experiment 1						
Ryegrass	30.0	25.7	56.3	23.5	--	70.4
Alfalfa hay	85.0	24.0	37.5	25.6	--	68.7
Leucaena	33.6	21.5	39.7	26.9	--	67.7
Corn silage	27.9	14.0	48.7	32.6	27.3	63.2
Spinifex	49.8	9.9	75.1	40.8	--	56.6
Buffel grass	40.4	9.5	64.2	37.8	--	59.0
Flinders grass	48.3	8.0	61.6	42.8	--	55.1
Mitchell grass	92.3	7.7	73.0	46.5	--	52.2
Rhodes grass hay	92.1	6.5	69.6	39.7	--	57.5
Queensland bluegrass	66.8	2.9	68.2	46.1	--	52.5
Experiment 2						
High-density barley^3^	90.5	11.7	--	--	42.2	84.0
Low-density barley^4^	90.5	13.0	--	--	41.1	82.0
Corn	89.6	8.7	--	--	72.5	88.0
Sorghum	89.8	11.5	--	--	55.1	74.0
Wheat	90.2	14.0	--	--	66.4	80.0

DM, dry matter; CP, crude protein; NDF, neutral detergent fiber; ADF, acid detergent fiber; TDN, total digestible nutrients.

Calculated according to the equations described by Holland and Kezar (1995) and by values obtained from NASEM (2016) for experiments 1 and 2, respectively.

High-density barley = 82 g/100 mL.

Low-density barley = 69 g/100 mL.

The experimental treatments evaluated were control (no probiotic inoculation; **CON**) or inoculation of a mixture of a DFM containing *Bacillus licheniformis* and *B. subtilis* (3.2 × 10^9^ CFU of the mixture per g; Bovacillus, Chr. Hansen A/S, Horsholm, Denmark; **DFM**) into the jars containing the in vitro media. The calculation of the dose to be incubated into each jar assigned to receive DFM was based on a rumen capacity of 70 L and the dose of 3 g of the DFM mixture per head per day.

#### Donor animals and inoculum collection.

Three rumen-fistulated Holstein steers were used as the inoculum source for the present study. The donor steers were fed ad libitum once daily a partial mixed ration (**PMR**) that contained (as-fed basis) 45% oat silage, 45% corn silage, and 10% concentrate mixture and grazed on a kikuyu paddock, with free access to water and mineral–vitamin mixture. The PMR did not contain any other nutritional additive such as prebiotics, probiotics, enzymes, ionophores, and non-ionophores (e.g., virginiamycin), for 10 d prior to rumen collection to ensure that the rumen environment and amylolytic bacteria would be present in the rumen fluid. Approximately 4 L of rumen fluid were pumped into the pre-heated thermos and immediately transported to the laboratory, where they were immediately flushed with CO_2_, blended, and squeezed through two layers of cheesecloth. Rumen fluids from the three steers were pooled into the thermos, at approximately equal amounts, for further laboratory analysis.

#### In vitro neutral detergent fiber digestibility procedure.

The in vitro NDFD procedure utilized a DAISY-II fermentation technique (ANKOM Technology Corp., Fairport, NY) modified to use the buffer and nutrient solution described by Goering and Van Soest (1970). All forage substrates were dried for 48 h in a forced-air oven at 60 °C and ground to pass a 2-mm screen in a Wiley mill. ANKOM F-57 filter bags (Ankom Technology, Macedon, NY) were used to isolate samples. Filter bags were prerinsed in a 95% acetone solution for 5 min to remove the surfactant that inhibits fermentation. Filter bags were air-dried, labeled with solvent-resistant marker pens, placed in a 105 °C oven for 1 h, transferred into a desiccator until it reached room temperature, and weighed. Samples (0.5 g) were then weighed into these filter bags, and incubated (6 replicates per sample) for either 24 or 48 h into 12 jars that could be inoculated with up to 20 bags per jar (6 jars per treatment). After incubation for 24 or 48 h, the bags were immediately placed in cold water to stop the microbial activity and rinsed under running water until the water became clear. Bags were dried at 60 °C in a forced-air oven for 48 h then weighed again for determination of IVDMD and then analyzed for aNDFom as described previously to determine both dry matter (**DM**) digestibility and neutral detergent fiber (**NDF**) digestibility. Blank samples (bags) were also included in the assay for further calculation and adjustment.

### Experiment 2: Concentrate Sources

This study was conducted at the University of Queensland (Gatton campus) from November 2021 to January 2022.

Five common cereal grains were collected and evaluated: high-density barley (82 g/100 mL), low-density barley (69 g/100 mL), corn, sorghum, and wheat. The nutritional profile of the substrates is presented in Table 1. The experimental treatments evaluated herein were the same as reported in experiment 1 (CON and DFM), following the same rationale for calculation of the dose to be incubated (3 g per head of a mixture containing *B. licheniformis* and *B. subtilis*; 3.2 × 10^9^ CFU of the mixture per g; Bovacillus, Chr. Hansen A/S) into the jars containing the rumen fluid of the animals.

#### Donor animals and inoculum collection.

Three rumen-fistulated Holstein steers were used as the inoculum source for the present study. The steers were fed a high-concentrate diet twice daily, without any other nutritional additive such as prebiotics, probiotics, enzymes, ionophores, and non-ionophores, for 10 d prior to rumen collection to ensure that the rumen environment and amylolytic bacteria would be present in the rumen fluid. In addition, steers were fed the diet ad libitum and the procedure for the collection followed the same procedures as described in experiment 1.

#### Incubation procedure.

For this starch digestion trial, prerinsed in acetone Ankom F-57 filter bags were used to isolate grain samples. Approximately 0.6 g of ground (1 mm screen) samples were weighed in each bag, sealed, shaken, and placed into the digestion bottles so that bags were evenly distributed on both sides of the divider of each digestion bottle with nine replicates for each sample. The fermentation procedure was similar to experiment 1 with IVDMD being determined and the residuals were analyzed for starch to determine IVSD.

#### Starch digestibility determination.

Total starch of grain and residuals after incubation were analyzed, in duplicate, using the Megazyme K-TSTA-100A total starch assay kit (Megazyme Ltd., Wicklow, Ireland) as per the instructions.

### Statistical Analysis

For both experiments, the jar was considered the experimental unit, and all the data were analyzed using the PROC MIXED procedure of SAS (Version 9.4; SAS Inst. Inc., Cary, NC, USA) and the Satterthwaite approximation to determine the denominator df for the test of fixed effects. For all analyses, the model statement for IVDMD, IVNDFD (experiment 1), and IVSD (experiment 2) contained the effects of treatment, hour (24 or 48 h for experiment 1; 6 or 12 h for experiment 2, respectively), and the resulting interaction. Data were analyzed using bag (jar), jar (treatment), and run as the random variables. The specified term for the repeated statement was an hour, the subject was bag (jar × treatment), and the covariance structure was first-order autoregressive, which provided the best fit for these analyses according to the smallest Akaike Information Criterion. Results are reported as least square means and were separated using the PDIFF structure. Furthermore, forage sources evaluated in experiment 1 were also classified as HIGH- or LOW-quality, based on the threshold value of 8.0% CP content (six forage sources into HIGH and four forage sources into LOW) and the effects of treatment were also evaluated using this quality classification. For all the data, significance was set at *P* ≤ 0.05 and tendencies were denoted if *P* > 0.05 and *P* ≤ 0.10. Results are reported according to the main effects if no interactions were significant.

## RESULTS

### Experiment 1

**Figure 1.**
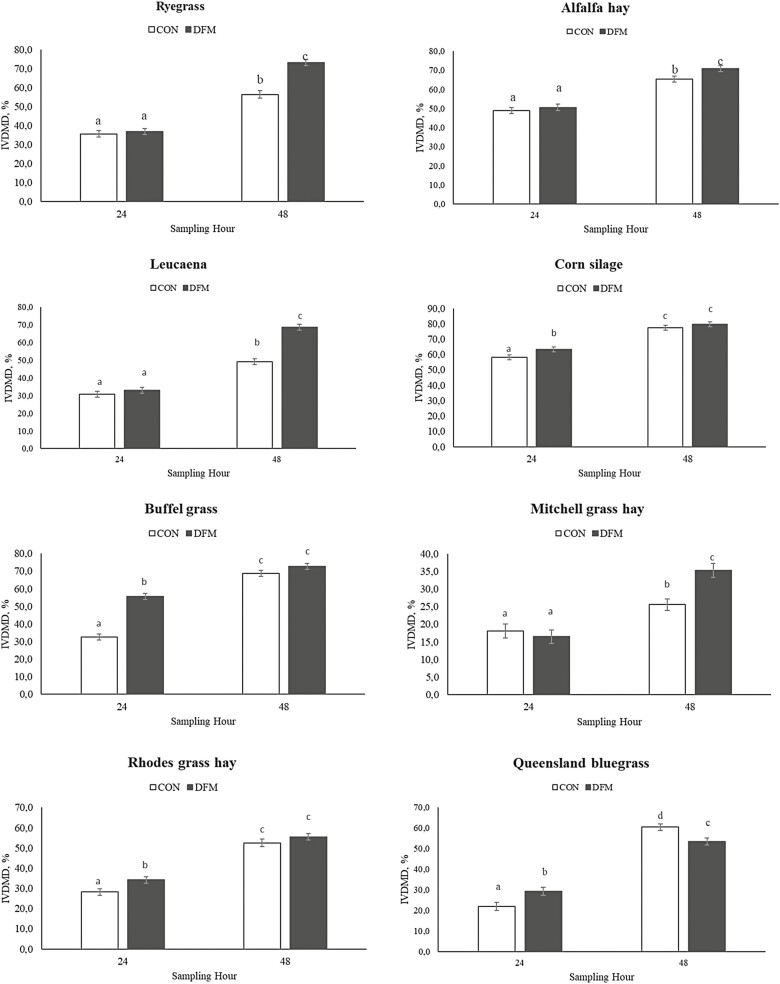
In vitro dry matter digestibility of forage sources evaluated at 24- and 48-h post-inoculation or not of a *Bacillus*-based direct-fed microbial. Forages reported herein had a significant treatment × hour interaction (*P* ≤ 0.05). Different letters indicate differences between treatments and hours (*P* ≤ 0.05).

Treatment × hour interactions were observed for IVDMD in 8 out of 10 forage sources (*P* ≤ 0.05). More specifically, DFM inoculation increased (*P* ≤ 0.03) IVDMD at 24 h in buffel grass, Queensland bluegrass, corn silage, and rhodes grass hay, an improvement that ranged from 8.5% to 70.8% (Figure 1). Moreover, 48-h IVDMD was also greater (*P* ≤ 0.02) following DFM inoculation for alfalfa hay (8.4%), ryegrass (29.8%), leucaena (39.9%), and Mitchell grass hay (38.2%), but opposite results were observed for Queensland bluegrass (11.5%; *P* < 0.01; Figure 1). Mean IVDMD was greater in 8 out of 10 forage sources following DFM inoculation (*P* ≤ 0.04), exception being Queensland bluegrass and flinders grass (*P* ≥ 0.60; Table 2).

**Table 2.** Mean in vitro dry matter digestibility of different forage sources inoculated or not with a *Bacillus*-based direct-fed microbial in experiment 1^1,2^

Forage source	Treatments		SEM	*P*-value^3^	
	CON	DFM		T	T × H
Ryegrass	46.0	55.1	1.21	<0.0001	<0.01
Alfalfa hay	57.1	60.7	1.11	0.03	0.05
Leucaena	39.9	50.8	1.14	<0.0001	<0.01
Corn silage	67.9	71.4	1.10	0.03	0.05
Spinifex	33.6	37.3	1.28	0.04	0.23
Buffel grass	50.7	64.2	1.14	<0.0001	<0.0001
Flinders grass	55.1	56.1	1.38	0.60	0.44
Mitchell grass	21.8	25.9	1.34	0.04	<0.01
Rhodes grass hay	40.4	44.9	1.21	0.01	0.04
Queensland bluegrass	41.1	41.3	1.84	0.93	0.03

IVDMD was analyzed at 24 and 48 h post-direct-fed microbial inoculation.

DFM, *Bacillus*-based direct-fed microbial inoculated in the rumen fluid (*B. licheniformis* and *B. subtilis*; Bovacillus, Chr. Hansen A/S, Horsholm, Denmark).

T, main treatment effect; T × H, treatment × hour interaction.

**Figure 2.**
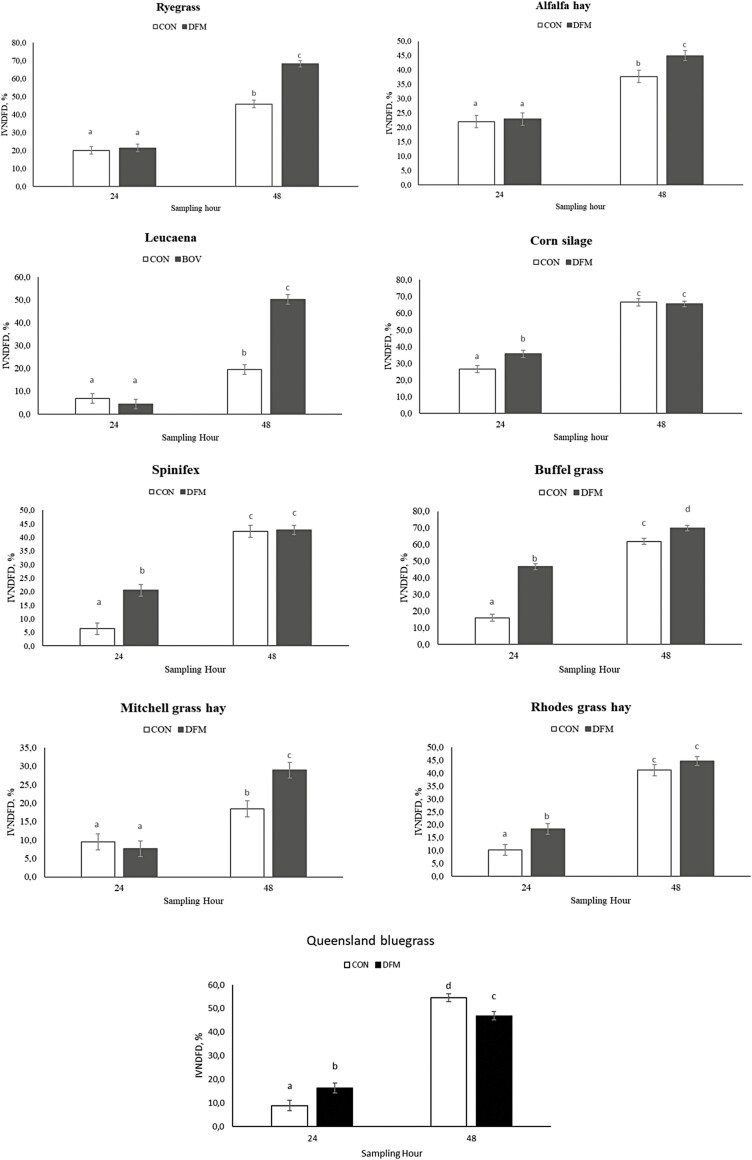
In vitro neutral detergent fiber digestibility of forage sources evaluated at 24- and 48-h post-inoculation or not of a *Bacillus*-based direct-fed microbial. Forages reported herein had a significant treatment × hour interaction (*P* ≤ 0.05). Different letters indicate differences between treatments and hours (*P* ≤ 0.05).

In a similar fashion, treatment × hour interactions were observed for IVNDFD in 9 out of the 10 forage sources evaluated herein (*P* ≤ 0.05). A 24-h IVNDFD increased following DFM inoculation (*P* ≤ 0.02) in spinifex, buffel grass, Queensland bluegrass, corn silage, and rhodes grass hay (Figure 2). DFM inoculation also improved 48-h IVNDFD of buffel grass, alfalfa hay, ryegrass, leucaena, and Mitchell grass hay (*P* ≤ 0.01), but reduced IVNDFD was observed for Queensland bluegrass (*P* < 0.01, Figure 2). Overall, the mean IVNDFD was greater in 8 out of 10 forage sources following DFM inoculation (*P* ≤ 0.05), the exception being Queensland bluegrass and flinders grass (*P* ≥ 0.23; Table 3).

**Table 3.** Mean in vitro neutral detergent fiber digestibility of different forage sources inoculated or not with a *Bacillus*-based direct-fed microbial in experiment 1^1,2^

Forage source	Treatments		SEM	*P*-value^3^	
	CON	DFM		T	T × H
Ryegrass	33.0	44.8	1.51	<0.0001	0.03
Alfalfa hay	29.9	34.0	1.38	0.04	0.04
Leucaena	6.32	22.9	1.38	<0.0001	<0.0001
Corn silage	46.6	50.6	1.45	0.05	0.04
Spinifex	24.2	33.1	1.44	<0.0001	0.05
Buffel grass	38.9	58.2	1.31	<0.0001	<0.0001
Flinders grass	42.4	44.8	1.38	0.23	0.88
Mitchell grass	14.0	18.3	1.52	<0.01	<0.0001
Rhodes grass hay	25.7	31.5	1.52	0.05	0.04
Queensland bluegrass	31.7	31.6	1.38	0.96	0.04

IVDMD was analyzed at 24 and 48 h post-direct-fed microbial inoculation.

DFM, *Bacillus*-based direct-fed microbial inoculated in the rumen fluid (*B. licheniformis* and *B. subtilis*; Bovacillus, Chr. Hansen A/S, Horsholm, Denmark).

T, main treatment effect; T × H, treatment × hour interaction.

When the forages were classified according to their quality by using CP as the threshold value, main treatment and forage quality effects were detected for IVDMD (*P* ≤ 0.02), whereas only main treatment effects were observed on IVNDFD (*P* < 0.01). No further effects or interactions were observed for both variables (*P* ≥ 0.13). The inoculation of the rumen fluid with DFM increased IVDMD and IVNDFD from 45.8% to 51.8% and from 29.3% to 37.4%, respectively.

### Experiment 2

**Figure 3.**
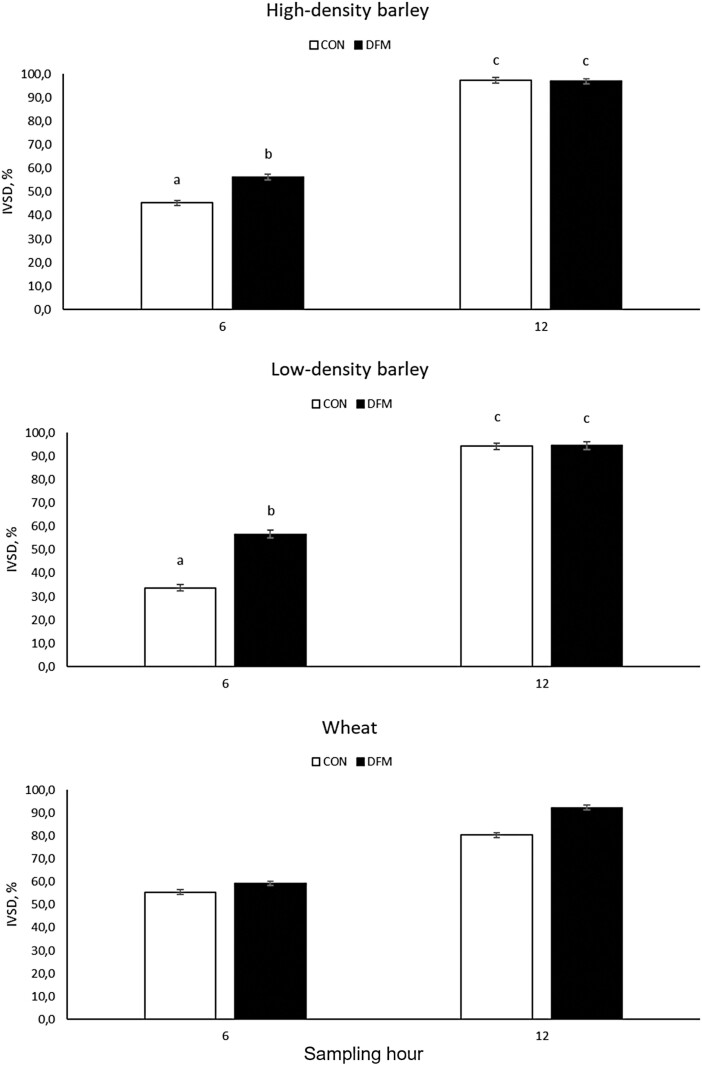
In vitro starch digestibility of concentrate sources was evaluated at 6- and 12-h post-inoculation or not of a *Bacillus*-based direct-fed microbial. Concentrates reported herein had a significant treatment × hour interaction (*P* ≤ 0.05). Different letters indicate differences between treatments and hours (*P* ≤ 0.05).

Treatment × hour interactions were observed for IVSD in three out of five concentrate sources evaluated herein (*P* ≤ 0.001), the exception being corn and sorghum that had the main treatment effect on IVSD (*P* ≤ 0.01). Inoculation of DFM into the in vitro system yielded greater IVSD for high-, low-density barley, and wheat (*P* ≤ 0.02), but also greater IVSD at 48 h in wheat (*P* < 0.0001; Figure 3). Moreover, the mean IVSD was also greater for corn and sorghum inoculated with DFM (*P* < 0.01; Table 4).

**Table 4.** Mean in vitro starch digestibility of different concentrate sources inoculated or not with a *Bacillus*-based direct-fed microbial in experiment 2^1,2^

Concentrate source	Treatments		SEM	*P*-value^3^	
	CON	DFM		T	T × H
High-density barley	71.3	76.3	0.80	<0.0001	<0.0001
Low-density barley	63.9	75.6	1.02	<0.0001	<0.0001
Corn	52.5	55.7	0.75	<0.01	0.41
Sorghum	37.0	42.0	0.91	<0.001	0.22
Wheat	67.9	75.6	0.54	<0.0001	<0.0001

IVSD was analyzed at 6 and 12 h post-direct-fed microbial inoculation.

DFM, *Bacillus*-based direct-fed microbial inoculated in the rumen fluid (*B. licheniformis* and *B. subtilis*; Bovacillus, Chr. Hansen A/S, Horsholm, Denmark).

T, main treatment effect; T × H, treatment × hour interaction.

## DISCUSSION

This article evaluates the effects of adding a *Bacillus*-based DFM on in vitro DM, aNDFom, and starch digestibility of a range of feeds. *Bacillus* spp. have been effectively utilized as probiotic microorganisms for human, poultry, and swine (Cutting, 2011; Luise et al., 2022). In ruminants, only a few studies evaluated the effects of such probiotics on performance, rumen fermentation profile, and health of calves (Sun et al., 2013; Deng et al., 2021; Lucey et al., 2021), beef steers (Colombo et al., 2021), or lactating dairy cows (Sun et al., 2013; Souza et al., 2017; Oyebade et al., 2021). Even fewer reports in the literature evaluated the combination of different *Bacillus* spp., such as *B. licheniformis* and *B. subtilis*. Kritas et al. (2006) reported an improvement in milk production (g/d), and milk fat and protein content (%) when a mixture of *B. licheniformis* and *B. subtilis* (1:1 ratio) was fed to pregnant ewes starting from 45 d pre- to 75 d post-lambing. In another trial, Kowalski et al. (2009) reported a greater average daily gain (+50 g/d), weaning body weight (+ 2.9 kg), and starter intake (+ 130 g/d) in Holstein calves supplemented with *B. licheniformis* and *B. subtilis* during the preweaning period.

In beef and dairy cattle, forages represent the major portion of the diets (Beauchemin et al., 2003; Alvarez et al., 2009), but the fiber level and type (cool- or warm-season) of these feedstuffs limit rumen digestibility and, consequently, herd productivity (Bohnert et al., 2011; Adesogan et al., 2014; Romero et al., 2016). One alternative to increase forage digestibility is to treat it with fibrolytic enzymes (Dean et al., 2005), but results have been variable (Beauchemin et al., 2003), mainly due to substrate type limiting the accessibility and, therefore, the efficacy of fibrolytic enzymes in hydrolyzing cellulose into glucose (Zhang et al., 2015). To mitigate this latter issue, expansin-like proteins, which can be encoded by several bacteria and fungi, can loosen, expand, or disrupt plant cell wall components, such as cellulose and hemicellulose (Liu et al., 2015). Recently, Pech-Cervantes et al. (2019) demonstrated that *B. subtilis* can produce and release expansin-like proteins in greater amounts than *Trichoderma reesei* (Liu et al., 2015). Moreover, hydrolytic activity of cellulase was increased when *B. subtilis* was incubated into a media vs. cellulase alone (Pech-Cervantes et al., 2019). In agreement with the latter authors, in experiment 1, mean IVDMD and IVNDFD increased by 13.1% (6.0 percentage points) and 27.6% (8.1 percentage points), respectively, following inoculation of a *Bacillus*-based DFM. Moreover, the treatment × hour interactions observed for most of the forages evaluated herein might be related to the forage type, DM, and nutrient content. Different Bacilli strains produce a different set of enzymes, in a manner that *B. licheniformis* synthesizes cellulases and *B. subtilis* produces expansin-like proteins (Pech-Cervantes et al., 2019; da Silva et al., 2021; Luise et al., 2022). When combined, these enzymes might have additive effects, as reported herein and also by Pech-Cervantes et al. (2019). In fact, Bunterngsook et al. (2014) observed greater synergism between expansin-like proteins and fibrolytic enzymes in substrates containing a higher proportion of hemicellulose, probably due to the breakage of hydrogen bonds between hemicellulose and cellulose, thereby increasing accessibility of cellulases to cell wall polysaccharides (Saloheimo et al., 2002). Nonetheless, when the quality of the forages was considered in the analysis of experiment 1, no differences were observed, suggesting that regardless of forage quality, improvements in IVDMD and IVNDFD were observed following inoculation of a *Bacillus*-based DFM.

NDF digestibility of forage sources is often limited by the cross-linking of lignin to other fibrous components (Jung et al., 2012). As reported by Oba and Allen (1999), every 1-unit improvement in NDF digestibility will increase DMI, milk yield, and 4% fat-corrected milk yield by 0.17, 0.23, and 0.25 kg, respectively, demonstrating that alternatives to improve NDF digestibility are imperative to improve the profitability of dairy cattle operations. It is important to mention that caution should be taken when analyzing and translating in vitro data into in vivo performance results, but the results from experiment 1 substantiate a possible NDFD improvement in the range of 3% to 4%, considering a dairy cow diet containing approximately 30% corn silage. This, in turn, would potentially lead to 0.75 to 1.0 L more fat-corrected milk per cow per day. Ferraretto et al. (2015) reported that hybrid selection for corn silage, for example, could be used as an alternative to promote a greater NDF digestibility of forage sources often fed to cattle. Recently, Pech-Cervantes et al. (2019) reported that inoculating *B. subtilis* with a fibrolytic enzyme increased NDF digestibility (+8.5%) of a dairy cow total mixed ration (**TMR**), supporting the results from experiment 1. Lastly, a greater NDF digestibility is likely to support a greater DMI of the herd, as reduced ruminal fiber disappearance and increased rumen fill inhibit DMI (Mertens, 1987; Ferraretto et al., 2013), which, in turn, will lead to greater milk production (Ferraretto et al., 2013).

Starch digestibility plays a key role in the performance of beef (Vander Pol et al., 2008; Owens et al., 2016) and dairy cattle (Ferraretto et al., 2013). Hence, it is imperative to maximize starch digestion in the rumen and, consequently, to increase total starch digestibility, reducing the amount of fecal starch in beef and dairy cattle (Ferraretto et al., 2013; Owens et al., 2016). Technologies to promote such improvements include grain processing (Owens et al., 1997, 2016; Marques et al., 2016) and utilization of enzymes, such as amylases (Mora et al., 2002). In dairy cattle, Ferraretto et al. (2011) reported minimal beneficial effects of feeding an exogenous amylase on milk production, composition, and efficiency of production in lactating Holstein cows.

Rojo et al. (2005) reported a greater activity of amylase produced by *B. licheniformis* when compared with a glucoamylase produced by *Aspergillus niger* or found in the rumen fluid from Holstein cows. Moreover, feeding amylases from *B. licheniformis* improved feed efficiency in sheep receiving a high-starch diet (70% sorghum) vs. control, and the feeding of amylases from *A. niger*, demonstrating that amylases from *B. licheniformis* could be considered a feasible alternative to improve starch digestion in the rumen of ruminants receiving a high-starch diet (Rojo et al., 2005). On average, the average improvement in IVSD following *B. licheniformis* and *B. subtilis* inoculation ranged from 6.0% (corn) to 18.2% (low-density barley). Among the feedstuffs that had a significant treatment × hour interaction, DFM inoculation increased IVSD at 6 h in the two barley types (low- and high-density) and wheat, whereas significant effects at 12 h were observed only for wheat. Main DFM effects on corn and sorghum suggest that there is a consistent greater starch digestibility of these sources, which, could be related to kernel vitreousness and their reduced starch digestibility compared with barley and wheat.

## CONCLUSION

Inoculating a *Bacillus*-based DFM (*B. licheniformis* and *B. subtilis*) improved mean in vitro DM and NDF digestibility of different forage sources of varying qualities (based on CP and NDF content). Similarly, IVSD of high- and low-density barley, corn, sorghum, and wheat was also greater following DFM inoculation, highlighting the potential of this probiotic to improve the nutrient digestibility and performance of the beef and dairy cattle herd. Nonetheless, additional studies are warranted to evaluate the effects of using a *Bacillus*-based DFM on in vitro and in situ digestibility of TMR often fed to the beef and dairy cattle herd, as well as additional performance trials that demonstrate the benefits of these probiotic strains into livestock production settings.
